# A Massive Liver Cyst Mimicking Decompensated Heart Failure: A Diagnostic Pitfall

**DOI:** 10.7759/cureus.101564

**Published:** 2026-01-14

**Authors:** Shandy Wong, Hayley Hong, Roshan M Lalmalani

**Affiliations:** 1 Geriatrics, Sengkang General Hospital, Singapore, SGP; 2 Family Medicine, SingHealth Polyclinics, Singapore, SGP

**Keywords:** : acute kidney injury, giant liver cyst, hypotension, liver cyst drainage, right atrium compression, right-sided heart failure

## Abstract

Simple hepatic cysts are common benign liver lesions that are typically asymptomatic. Although complications such as intracystic hemorrhage or rupture may occur, they are rare. Mass effect is more likely to be seen in cases of markedly enlarged cysts, which may compress adjacent organs and lead to atypical clinical presentations. We report a rare case of a massive hepatic cyst causing severe external compression of the right heart, resulting in hypotension, oliguric acute kidney injury, and critical limb ischemia. Percutaneous drainage of the cyst led to immediate hemodynamic improvement, with rapid resolution of hypotension and dramatic recovery of renal function and urine output. This case highlights an unusual presentation of a giant hepatic cyst, discusses the associated diagnostic challenges, and reviews important considerations in its evaluation and management.

## Introduction

Simple hepatic cysts are benign liver tumors, with an estimated prevalence ranging from 5% to 10%, of which only 5% of cases report complications [1]. While often asymptomatic, with most cases incidentally discovered through imaging, hepatic cysts can lead to rare intracystic complications such as intracystic hemorrhage, infection, and cyst rupture. Other hepatic complications include both intrahepatic and extrahepatic obstruction to the biliary system, leading to obstructive jaundice and risk of acute cholangitis [2]. Additionally, patients may experience symptoms due to the compression of enlarging hepatic cysts on neighboring intraperitoneal and intrathoracic organs. These symptoms may include abdominal distension, gastroesophageal reflux, early satiety, severe malnutrition [3], dyspnea, reduced exercise tolerance, and back pain.

As the liver and heart are closely related, cardiac complications resulting from hepatic cysts have been reported infrequently [3]. Case reports on cardiac complications are rare and may include presentations such as arrhythmias, hemodynamic instability, or even sudden cardiac death. Another case reports compression on the inferior vena cava by a large hepatic cyst, leading to inferior vena cava thrombosis [4]. In this article, we present an unusual case of a patient with a large simple hepatic cyst who exhibited signs and symptoms of right heart failure with hypotension due to external compression of the right atrium and ventricle.

## Case presentation

An 87-year-old Chinese female patient, known to have a hepatic cyst and choledocholithiasis with no other chronic medical conditions, presented to the emergency department with a one-week history of worsening bilateral lower limb swelling and mild abdominal bloating. She denied experiencing symptoms such as chest pain, shortness of breath, orthopnea, paroxysmal nocturnal dyspnea, diarrhea, nausea, and vomiting. There was no fever or abdominal pain reported. She was not on any chronic medications and had no recent travel history.

On inspection, a thin and lethargic patient was observed. She was afebrile, with a blood pressure of 87/61 mmHg, a pulse rate of 98 beats per minute, and an oxygen saturation of 97%. Cardiac examination revealed a pansystolic murmur heard loudest over the axilla, along with the presence of pitting edema over the sacral region and in her lower limbs extending up to the thighs bilaterally. The jugular venous pressure was not raised, and bilateral lung fields were clear on auscultation, with equal air entry. Her extremities were cold to the touch, with a poorly palpable left dorsalis pedis pulse. The abdomen was distended but otherwise soft and nontender.

Pertinent findings from the initial laboratory investigations reveal elevated serum alkaline phosphatase (ALP) levels of 498 IU/L and serum gamma-glutamyl transferase (GGT) levels of 658 IU/L. Additionally, there was a gradual development of acute kidney injury, evidenced by the rise in serum creatinine from 76 mL/min to 171 mL/min over a one-week duration with worsening urine output. This was accompanied by worsening lactic acidosis. Elevated inflammatory markers were also observed, including a serum C-reactive protein level of 107 mg/dL, serum procalcitonin of 1.91 μg/L, and a white blood cell count of 13.95 x 10^9^/L. There was no eosinophilia. Blood cultures returned negative.

Considering her history of choledocholithiasis, rising inflammatory markers, and an obstructive picture on her liver function tests, she was initially treated for hepatobiliary sepsis with concomitant heart failure and acute kidney injury. She was started on intravenous antibiotics and diuretics. However, she responded poorly to treatment with worsening renal function and persistently deranged liver function tests. Additionally, she displayed signs of critical limb ischemia as evidenced by right lower limb rest pain, poorly felt lower limb pulses, cool peripheries, and rising serum lactate.

**Figure 1:**
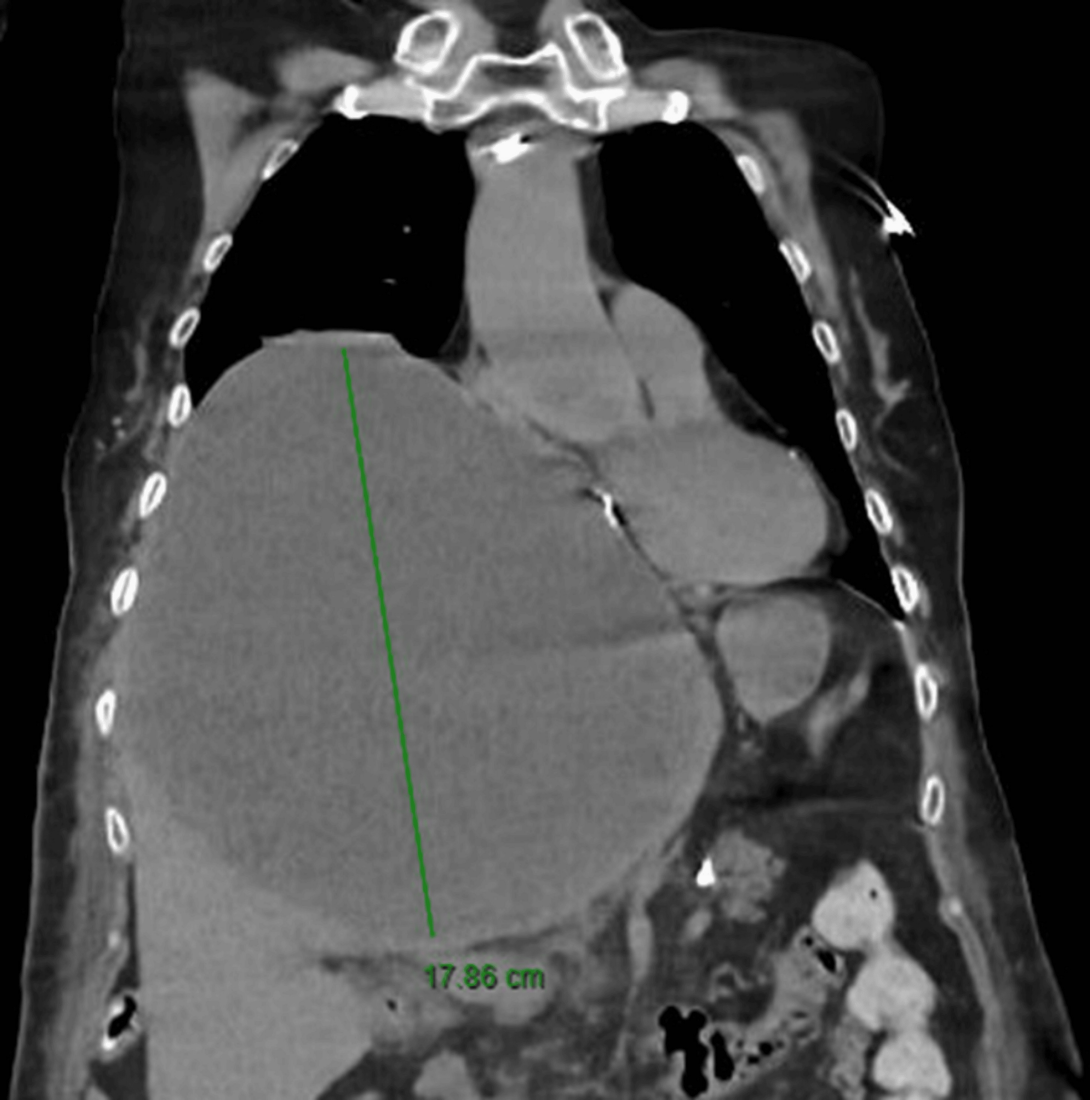
Coronal section of CT images displaying resultant mediastinal shift secondary to a large hepatic cyst measuring 17.86 cm

**Figure 2:**
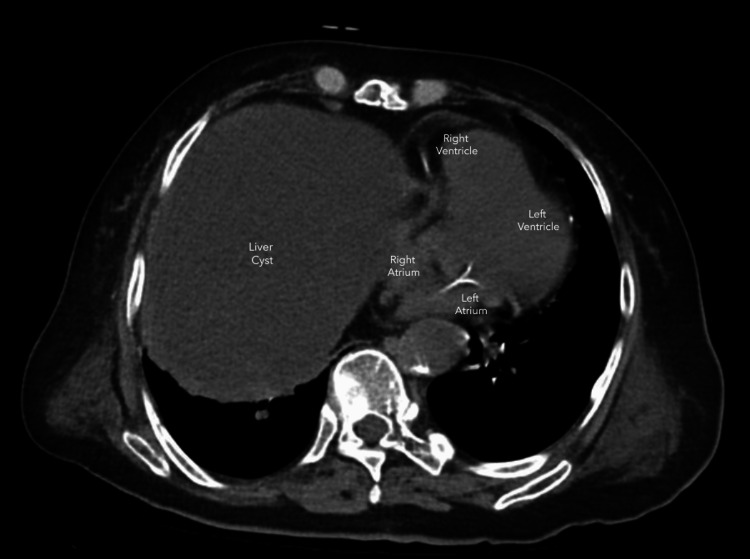
Axial cut of CT images showing extrinsic compression of the right heart by a large simple hepatic cyst

Several imaging studies were conducted based on the preliminary assessment. A computed tomography (CT) scan of the abdomen and pelvis demonstrated a large septate hepatic cyst centered in the left lobe, measuring approximately 18.5 cm by 12.4 cm by 17.7 cm (Figure 1). This cyst caused elevation of the right hemidiaphragm and exerted mass effect on adjacent structures, including the right heart (Figure 2). Comparing this to old scans done previously, it is worth noting that there was a significant increase in size of the hepatic cyst compared to the dimensions of 11.2 cm by 6.3 cm by 8.5 cm noted on scans two years ago. There was otherwise no dilation of the biliary tree. There was no suggestion of intracystic hemorrhage on the scan.

**Figure 3:**
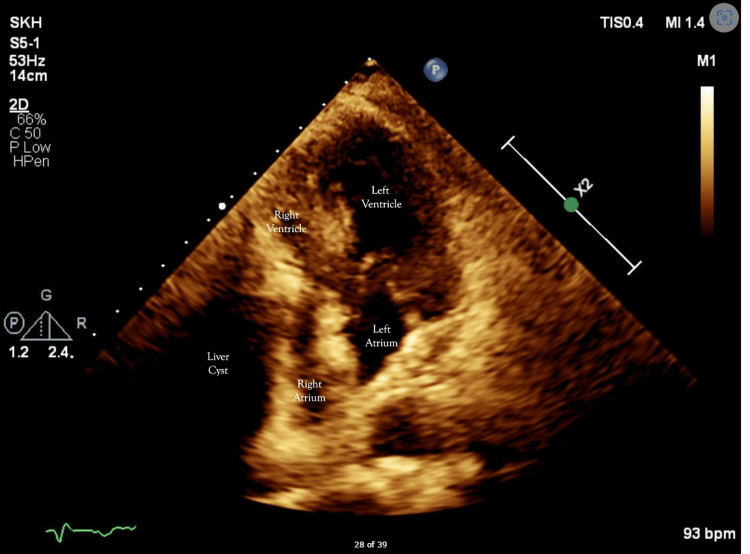
Transthoracic echocardiogram demonstrating compression of the right heart by the adjacent large liver cyst (four-chamber view)

Significantly, a transthoracic echocardiogram showed extrinsic compression of the right atrium and right ventricle (Figure 3). Otherwise, the left ventricular ejection fraction was 55%-60%, with no regional wall motion abnormality. Incidentally, there was mild to moderate mitral regurgitation.

**Figure 4:**
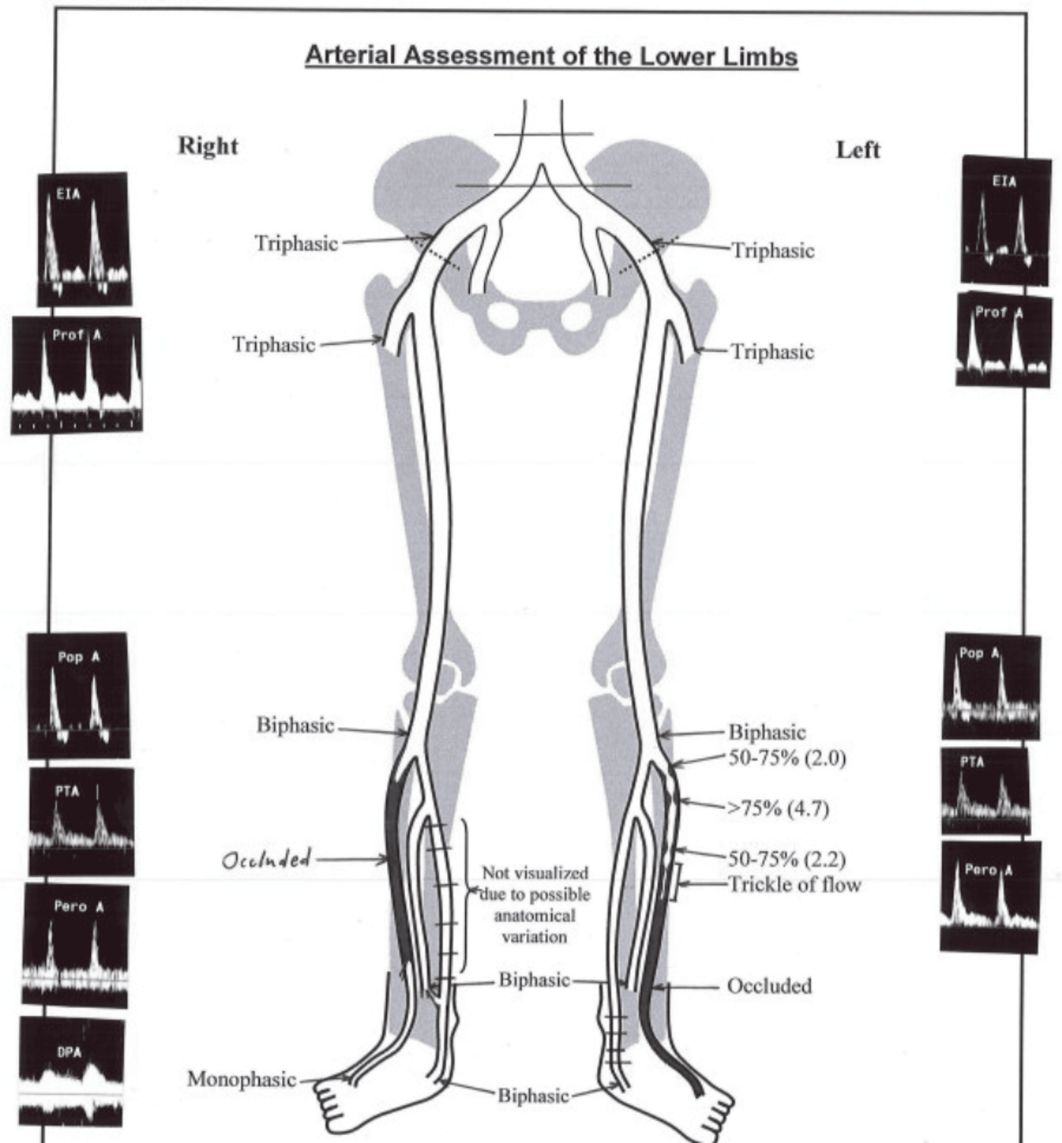
Arterial duplex ultrasound of bilateral lower limbs showing chronic occlusion of bilateral anterior tibial arteries

An arterial duplex ultrasound study of the lower limbs revealed chronic occlusion in bilateral anterior tibial arteries (Figure 4), although this was deemed unlikely to be the cause of her lactic acidosis given the presence of collateral circulation.

The constellation of signs and symptoms exhibited by our patient led to the hypothesis of right heart failure secondary to extrinsic compression by the large hepatic cyst. This condition resulted in lower limb swelling, an obstructive pattern of liver function abnormality, and the sequelae of reduced left ventricular output. This includes hemodynamic instability, acute kidney injury, and lower limb ischemia with lactic acidosis due to reduced tissue perfusion on a background of peripheral arterial disease [5].

Subsequently, the patient underwent ultrasound-guided percutaneous drainage of the hepatic cyst, leading to a rapid improvement clinically, as evidenced by improvements in blood pressure, lower limb swelling, and an increase in urine output. Biochemically, improvements in renal function, liver function, and lactic acidosis were observed (Table 1).

**Table 1:** Clinical and biochemical parameters before and after percutaneous drainage of the large hepatic cyst

	Before drainage	Day 1 post-drainage	Day 2 post-drainage	Day 3 post-drainage
Blood pressure (mmHg)	83/63	103/62	113/70	126/73
Urine output (ml/day)	275	634	2523	1130
Serum creatinine (umol/L)	201	181	122	78
Serum lactate (mmol/L)	5.0	2.2	1.7	-
Bilirubin (umol/L)	47	34	33	-
Alkaline phosphatase (U/L)	453	256	235	-

Following drainage of the liver cyst, a repeat ultrasound of the hepatobiliary system revealed an interval decrease in size of the liver cyst to 5.4 cm x 4.1 cm x 2.6 cm. A transthoracic echocardiogram was also repeated, which showed a normal right atrium and normal right ventricular systolic function. The drainage catheter was removed thereafter. Cytology report of the hepatic fluid revealed no malignant cells. The patient was offered subsequent sclerotherapy as definitive treatment, but she declined further procedures. 

## Discussion

The underlying pathogenesis of hepatic cysts is not fully understood. Most simple cysts are thought to be congenital and often arise from hyperplastic biliary ducts that lack communication to the biliary system [6]. Hepatic cysts are most often detected incidentally, with only a small fraction of them presenting with symptoms. They are seldom associated with life-threatening complications.

Furthermore, cardiac complications from hepatic cysts are rare, with the majority of the cases arising from hepatic cysts in the setting of polycystic liver disease. Reports of cardiac complications from simple hepatic cysts are few, and they commonly present with right heart failure. Algın et al. described a case of hemodynamic instability secondary to a polycystic liver disease, which showed rapid clinical improvement following cyst drainage [3]. Other uncommon cardiac complications reported include arrhythmias, where Ker reports a case of a large simple hepatic cyst with right atrial compression presenting as frequent atrial premature beats [7], and cardiac tamponade as described by Vives et al. [8]. Similarly, Dimek et al. report a case of symptomatic massive liver cyst presenting as a sudden cardiac arrest in a patient with no prior ischemic heart disease, before a planned surgical fenestration. In this case, a computed tomography scan of the coronary arteries did not reveal any significant coronary artery disease, while a cardiac magnetic resonance imaging scan done showed right atrial and ventricular compression by the large hepatic cyst throughout the cardiac cycle [9].

We present a patient with a unique constellation of symptoms of right heart failure, hemodynamic instability, acute kidney injury, and acute limb ischemia due to compressive symptoms of a large simple hepatic cyst. The presence of asymptomatic choledocholithiasis and raised white blood cell count was a red herring. In retrospect, elevated inflammatory markers may have been explained by the presence of acute limb ischemia. Marked clinical improvement was observed following percutaneous drainage of the large hepatic cyst, highlighting the importance of early diagnosis and timely intervention. 

In symptomatic simple hepatic cysts, treatment options include minimally invasive procedures such as percutaneous aspiration with subsequent sclerotherapy to reduce the chances of recurrence. Surgical fenestration via open or laparoscopic techniques is an alternate treatment option and is often associated with a lower risk of recurrence [6]. In complicated cases, a complete surgical resection of the liver cyst may be considered [10].

## Conclusions

Although simple hepatic cysts are common and typically asymptomatic, this case highlights that large cysts may give rise to a wide spectrum of complications. In patients presenting with atypical clinical features in the presence of a large hepatic cyst, clinicians should consider the cyst itself as a potential source of these complications.

While rare, cardiac complications of large hepatic cysts are potentially life-threatening. Large hepatic cysts exhibiting signs of cardiac compression should be treated with utmost seriousness, warranting prompt surgical intervention. Early diagnosis and intervention are critical, as large simple liver cysts can be readily managed with minimally invasive percutaneous aspiration or surgical fenestration, often yielding immediate and good relief of their compressive effects and symptoms. Given that this patient presented with a multitude of medical and surgical issues, including right heart failure, acute kidney injury, and critical limb ischemia secondary to a large hepatic cyst, it highlights the importance of a multidisciplinary approach in managing patients with large symptomatic liver cysts to achieve optimal outcomes in patients with large symptomatic hepatic cysts.
